# Study of Liver Dysfunction in Hyperemesis Gravidarum

**DOI:** 10.7759/cureus.8709

**Published:** 2020-06-20

**Authors:** Nayana Gaba, Saurabh Gaba

**Affiliations:** 1 Obstetrics and Gynaecology, Postgraduate Institute of Medical Education and Research, Chandigarh, IND; 2 General Medicine, Government Medical College and Hospital, Chandigarh, IND

**Keywords:** hyperemesis gravidarum, nausea and vomiting in pregnancy, liver, hepatic, liver function tests, liver dysfunction

## Abstract

Introduction

Hyperemesis gravidarum (HG) is said to occur when early pregnancy is complicated by excessive vomiting that leads to electrolyte imbalance, ketosis or loss of more than 5% of the bodyweight. It can be accompanied by deranged liver function tests (LFT), and most patients recover uneventfully with no fetal harm.

Methods

A retrospective study was conducted by evaluating records of 135 patients who were admitted or underwent day care for HG at our center over a period of 30 months. After excluding patients who were not investigated and those with another pre-existing or newly diagnosed liver disease, 63 patients were enrolled in the study. Their LFT were analyzed with the software Graphpad Prism version 8.4 (GraphPad Software, San Diego, California). The values were expressed as mean ± standard deviation and statistical analysis was done using unpaired t test and simple linear regression.

Results

The mean age of the study population was 26.59 ± 5.15 years and the mean period of gestation was 13.27 ± 2.48 weeks. 60.3% (38/63) of the patients had some form of abnormality on the LFT. The mean total serum bilirubin (TSB) was 1.56 ± 0.84 mg/dL, mean aspartate transaminase (AST) was 46.63 ± 30.89 U/L and mean alanine transaminase (ALT) was 51.35 ± 42.86 U/L. ALT was higher than AST with statistical significance (p<0.0001). There was no statistically significant difference in the LFT of primigravida and multigravida women. The study population included three diabetic and two hypertensive women, and two women had multiple pregnancy. All the patients were treated with anti-emetics. One patient required corticosteroid administration, and none required termination of pregnancy.

Conclusion

Mild liver dysfunction in HG can occur in over 50% of the patients. When diagnosis is not in doubt, no further intervention is required with regard to the LFT.

## Introduction

Hyperemesis gravidarum (HG) refers to the state of severe and intractable vomiting occurring in early pregnancy. It may be considered a part of the spectrum of nausea and vomiting that accompanies pregnancy and is diagnosed when vomiting is accompanied by fluid and electrolyte imbalance, ketosis or loss of more than 5% of the bodyweight [1]. Practically, diagnosis can be made when patients present with exaggerated symptoms of morning sickness, and alternate diagnoses have been excluded.

It has been found to complicate 0.3%-2.0% of all pregnancies [2]. Symptoms usually start in the late first trimester and most of the women show resolution after 20 weeks of gestation. The risk factors include first pregnancy, past history of HG, multiple pregnancy, gestational trophoblastic disease, and obesity [1].

The exact pathogenesis is still under investigation, but a causal relationship has been hypothesized with increased levels of human chorionic gonadotropin (hCG) [2]. This stems from the finding of a correlation between serum hCG level and vomiting. Moreover, conditions with increased hCG, such as multiple pregnancy and gestational trophoblastic disease, have an increased incidence of HG. The historically presumed association of HG with estrogen and progesterone has now been discredited beyond doubt [3]. In a large prospective study, the incidence of gastric Helicobacter pylori infection was found to be higher in pregnant women who had severe vomiting, but this is likely an altogether distinct condition, and plays no role in the pathogenesis of HG [4].

Mild nausea and vomiting can be managed by avoidance of triggers such as fatty foods, strong odors, and iron supplements, by using ginger containing foods and syrups, and by oral medications such as pyridoxine, doxylamine succinate, diphendydramine and metoclopramide [5]. For hyperemesis, admission may be required and management of fluid and electrolyte balance is vital. Oral medications are seldom tolerated and intravenous forms of diphendhydramine, metoclopramide, or ondansetron may be used. These are usually accompanied by thiamine and gastric acid suppressive medications such as ranitidine and omeprazole. Refractory cases may be treated with a short course of glucocorticoids such as methylprednisolone and hydrocortisone. Termination of pregnancy is the last resort, but it is rarely required [5]. For prolonged illness, nutritional support may have to be instituted. It can be given enterally through a Ryle’s tube, or parenterally if it is not tolerated [6].

The complications of HG are dehydration, hypokalemia, hypomagnesemia, hypocalcemia, hypochloremic metabolic alkalosis, abnormal liver function tests (LFT), pre-renal acute kidney injury, and transient hypothyroidism [2,7]. In the absence of severe malnutrition, the outcome is excellent with vomiting rarely persisting beyond 20 weeks of gestation [8]. There is no adverse effect on the fetal growth, and maternal nutritional status is corrected soon after normal feeding is resumed. With proper management, maternal mortality does not occur, although morbidity can result from some exceedingly rare complications such as Mallory Weiss tear of the esophagus, acute tubular necrosis, Wernicke’s encephalopathy, rhabdomyolysis and pneumothorax [9-11].

## Materials and methods

A retrospective study involving 63 patients who presented to our center (Government Medical College and Hospital, Chandigarh, India) over a period of 30 months was conducted. The records of all the pregnant patients with HG who required admission or day care were assessed. Out of a total of 135 patients, 65 did not undergo investigations, and another seven patients had a known or newly diagnosed liver disease, so they were excluded. The maximum recorded values of total serum bilirubin (TSB), aspartate transaminase (AST) and alanine transaminase (ALT) were used for analysis. Statistical analysis was carried out using the software Graphpad Prism version 8.4 (GraphPad Software, San Diego, California).

## Results

The chief observations have been summarized in Tables 1-2. 52.4% (33/63) of the patients had elevated TSB (>1.2 mg/dL), 41.3% (26/63) had elevated AST (>40 U/L), and 39.7% (25/63) had elevated ALT (>40 U/L). Overall, 60.3% (38/63) of the patients had some form of abnormality on the LFT. There was no statistically significant difference of LFT between the primigravida and the multigravida women (Table 3). The patients were managed with intravenous fluids and antiemetics. One patient required corticosteroid administration, and none required termination of pregnancy.

**Table 1:** Characteristics of the study population. SD: standard deviation; CI: confidence interval; TSB: total serum bilirubin; AST: aspartate transaminase; ALT: alanine transaminase.

Variable	Mean ± SD	95% CI	Range
Age (years)	26.59 ± 5.15	25.29 to 27.88	18-42
Period of gestation (weeks)	13.27 ± 2.48	12.64 to 13.90	9-21
TSB (mg/dL)	1.56 ± 0.84	1.35 to 1.78	0.3-3.9
AST (U/L)	46.63 ± 30.89	38.86 to 54.41	19-159
ALT (U/L)	51.35 ± 42.86	40.56 to 62.14	20-207

**Table 2:** Liver function tests of the study population. TSB: total serum bilirubin; AST: aspartate transaminase; ALT: alanine transaminase.

Investigation	Range	Number of patients
TSB (mg/dL)	≤1.2	30 (47.6%)
	1.3-2.0	19 (30.2%)
	2.1-3	10 (15.8%)
	3.1-4	4 (6.3%)
	>4	0
AST (U/L)	≤40	37 (58.7%)
	41-100	20 (31.7%)
	101-200	6 (9.5%)
	201-300	0
	>300	0
ALT (U/L)	≤40	38 (60.3%)
	41-100	19 (30.1%)
	101-200	5 (7.9%)
	201-300	1 (1.6%)
	>300	0

**Table 3:** Comparison of the liver function tests of primigravida and multigravida women. TSB: total serum bilirubin; AST: aspartate transaminase; ALT: alanine transaminase. Two-tailed p value was calculated using unpaired t test.

Investigation	Primigravida (n = 46)	Multigravida (n = 17)	P value
TSB (mg/dL)	1.57 ± 0.84	1.54 ± 0.88	0.8932
AST (U/L)	48.70 ± 32.94	41.06 ± 24.49	0.3880
ALT (U/L)	50.96 ± 40.73	52.41 ± 49.50	0.9059

**Figure 1:**
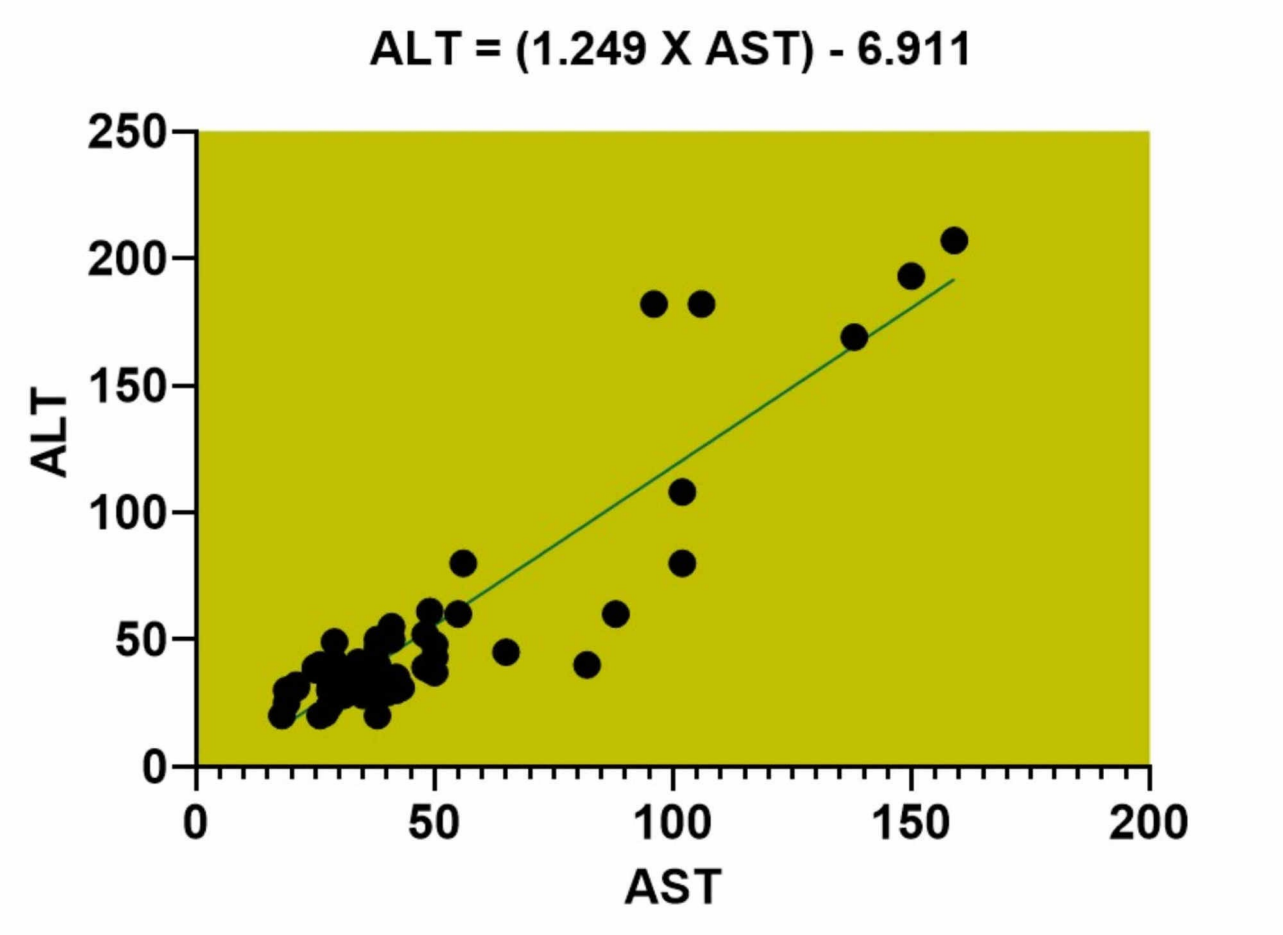
Graph depicting the simple linear regression analysis of AST and ALT of the study population. AST: aspartate transaminase; ALT: alanine transaminase.

Simple linear regression model was utilized to assess the association between AST and ALT (Figure 1). ALT was higher than AST with statistical significance (p<0.0001).

The study population included three diabetic women. They required temporary discontinuation of insulin and/or metformin, and none of them had hypoglycemia or ketoacidosis. Another two patients were hypertensive and one of them required temporary cessation of the antihypertensive therapy. Only two patients had multiple pregnancy, so no statistical conclusion could be drawn with respect to those with single pregnancy.

## Discussion

Liver disease in pregnancy has conventionally been divided into pre-existing disease, new-onset disease and pregnancy-specific disease (Table 4) [12]. Since a normal pregnancy is not accompanied by any significant changes in the hepatic physiology, the management of liver diseases not specific to pregnancy is mostly the same as general population [13]. Furthermore, normal pregnancy has no effect on AST and ALT, while there is a rise in alkaline phosphatase (ALP) and fall in serum albumin. Any abnormality observed in the transaminases needs to be evaluated.

**Table 4:** Classification of liver diseases in pregnancy. HELLP: hemolysis, elevated liver enzymes and low platelets.

Pregnancy-specific	Not pregnancy-specific (pre-existing or new-onset)
Acute fatty liver of pregnancy	Acute and chronic viral hepatitis
Pre-eclampsia	Alcohol and hepatotoxic drugs
Eclampsia	Non-alcoholic fatty liver disease
Intrahepatic cholestasis of pregnancy	Autoimmune hepatitis
HELLP syndrome	Metabolic disorders such as hemochromatosis, Wilson’s disease and glycogen storage diseases
Hyperemesis gravidarum	Budd-Chiari syndrome

Acute fatty liver of pregnancy (AFLP) is a rare disorder characterized by fatty infiltration of the liver in peripartum period, and it can progress to fulminant liver failure [14]. Hence, it requires early recognition and prompt termination of pregnancy. It complicates 0.01% of all pregnancies. The hepatic aminotransferase levels can rise to ten times the upper limit and deep jaundice can occur [12].

Liver involvement is common in the hypertensive disorders of pregnancy. Pre-eclampsia and eclampsia occur after twenty weeks of gestation. They can lead to hepatocellular necrosis with stark elevations of the aminotransferases [12]. Elevation to more than 100 times the upper limit is not uncommon, but jaundice is usually mild. Hepatic hemorrhage, rupture and infarction can also occur [15]. Management involves quick delivery of the fetus along with medical treatment of high blood pressure and seizures. Syndrome of hemolysis, elevated liver enzymes and low platelets (HELLP) is a variant of pre-eclampsia.

Intrahepatic cholestasis of pregnancy is ordinarily a benign condition complicating up to 5% of all pregnancies [12]. Presence of intrahepatic canalicular bile plugs is the pathological hallmark and pruritus is the main clinical feature. The elevation of aminotransferases and bilirubin is mild, but alkaline phosphatase is disproportionately elevated, sometimes reaching values above 1000 U/L. At least thirty-fold elevation of serum bile acids is seen. Management involves the use of ursodeoxycholic acid and anti-pruritic agents, and delivery as soon as fetal maturity is attained [16].

Liver involvement is mild and subclinical in HG. As a result, most authors don’t consider it a liver disease per se [12]. Studies have shown that derangement in LFT occurs in half of the patients with HG, and the biochemical abnormalities resolve on cessation of vomiting [17]. The findings of our study are in agreement with the data available. The aminotransferases can usually rise to twice the upper limit, and values more than 200 U/L are rarely recorded [12,18]. ALT has been observed to rise more than AST, but the reason for this is not known [19]. Rarely, mild jaundice can occur and bilirubin can rise up to 4 mg/dL. The degree of derangement of the LFT may correlate with the severity of vomiting, and their persistence after the cessation of vomiting should provoke further investigations to determine the underlying pathology. The perturbations of LFT in HG have no clinical impact on the hepatic functioning. The synthetic function remains intact, as evidenced by the absence of an effect on the coagulation profile and serum albumin levels, except when malnutrition occurs [13]. In an unusual case reported by Larrey et al., the patient developed HG in three consecutive pregnancies [20]. She had jaundice in all of them and the maximum recorded aminotransferase values were 22 times the upper limit of normal.

HG does not lead to any radiological changes in the liver. Ultrasound of the abdomen is commonly performed to rule out other causes of vomiting, such as cholecystitis and pancreatitis. It does not reveal any changes in the liver size, parenchymal echotexture or the biliary radicles. Any changes observed can be ascribed to other disorders rather than HG [13]. Liver biopsy is hardly ever indicated, but may be necessary when diagnosis is doubtful and atypical pattern of LFT is seen. The histopathology is either normal, or it may reveal some foci of hepatocelluar necrosis and centrilobular steatosis with occasional bile plugs [20].

The pathogenesis of hepatic dysfunction in HG is not fully understood. There is evidence of starvation and malnutrition leading to hepatic transaminase elevation but this does not explain its occurrence when the nutritional status is not compromised [21]. Kaplan et al. found that the maternal level of tumour necrosis factor alpha (TNF-α) was higher in patients with HG [22]. One of the sources of TNF-α is the placenta. This inflammatory cytokine may play a role in the pathogenesis of HG and concurrent liver dysfunction. In mouse models, TNF-α has been shown to induce apoptosis of T lymphocytes in liver with consequent injury [23]. Impaired metabolism of fatty acids that have accumulated in the placenta due to reduced fatty acid oxidation by the fetus or mother leads to production of reactive oxygen species (ROS) [24]. These ROS can increase the production of inflammatory cytokines by the placenta and cause HG and maternal liver damage. This mechanism plays role when there are defects in the fetal or maternal enzymes involved in oxidation of fatty acids.

 It is possible to have subclinical undiagnosed liver disease which can lead to isolated rise of the hepatic enzymes. This has the potential to confound the results. This issue can be addressed by conducting a longitudinal study wherein the LFT are also monitored before the development of HG and after its resolution. To the best of our knowledge, no such study has been conducted till date. Even if such a study is undertaken, the current understanding of the topic is not likely to change meaningfully. In addition, not all patients with HG are investigated. Patients with severe or complicated illness, and those with diagnostic uncertainty are usually the ones who are investigated. This shortcoming can also be overcome by a planned prospective study. The association of degree of liver dysfunction with the severity of illness is difficult to establish due to the lack of well-defined clinical criteria for severity. Recently, pregnancy-unique quantification of emesis and nausea (PUQE) scoring system has been validated for defining the severity of nausea and vomiting in pregnancy (NVP) on basis of the symptoms, but it is not widely used (Table 5) [25]. Moreover, it has no implication on the treatment and does not predict the course of HG.

**Table 5:** Pregnancy-unique quantification of emesis and nausea (PUQE) score for nausea and vomiting in pregnancy (NVP). Total points ≥6 in mild NVP, 7-12 in moderate NVP and ≥13 in severe NVP.

	5 points	4 points	3 points	2 points	1 point
Duration of nausea in a day (hours)	>6	4-6	2-3	≤1	No nausea
Number of vomitings in a day	≥7	5-6	3-4	1-2	No vomiting
Number of retchings in a day without bringing up food	≥7	5-6	3-4	1-2	No retching

## Conclusions

HG can lead to liver dysfunction in over half of the patients suffering from it. The LFT abnormalities are mild and inconsequential with regard to the outcome. No specific intervention is required; however, atypical patterns may necessitate other investigations to rule out another underlying disease.
